# Luminescent Amorphous Silicon Oxynitride Systems: High Quantum Efficiencies in the Visible Range

**DOI:** 10.3390/nano13071269

**Published:** 2023-04-03

**Authors:** Pengzhan Zhang, Leng Zhang, Fei Lyu, Danbei Wang, Ling Zhang, Kongpin Wu, Sake Wang, Chunmei Tang

**Affiliations:** 1 College of Electronic and Information Engineering, Jinling Institute of Technology, Nanjing 211169, China; 2 Collaborative Innovation Center of Advanced Microstructures, National Laboratory of Solid-State Microstructures, Nanjing University, Nanjing 210093, China; 3 College of Science, Hohai University, Nanjing 210098, China

**Keywords:** a-SiN_x_O_y_, photoluminescence quantum efficiency, integration sphere, temperature-dependent PL, PL mechanisms

## Abstract

In recent years, researchers have placed great importance on the use of silicon (Si)-related materials as efficient light sources for the purpose of realizing Si-based monolithic optoelectronic integration. Previous works were mostly focused on Si nanostructured materials, and, so far, exciting results from Si-based compounds are still lacking. In this paper, we have systematically demonstrated the high photoluminescence external quantum efficiency (PL EQE) and internal quantum efficiency (PL IQE) of amorphous silicon oxynitride (a-SiN_x_O_y_) systems. Within an integration sphere, we directly measured the PL EQE values of a-SiN_x_O_y_, which ranged from approximately 2% to 10% in the visible range at room temperature. Then, we calculated the related PL IQE through temperature-dependent PL measurements. The obtained PL IQE values (~84% at 480 nm emission peak wavelength) were very high compared with those of reported Si-based luminescent thin films. We also calculated the temperature-dependent PL EQE values of a-SiN_x_O_y_ systems, and discussed the related PL mechanisms.

## 1. Introduction

With the development of information technology, the communication industry urgently needs higher speeds, higher capacities, and lower power waste information transmission and processing. Since most current integrated circuits are manufactured on Si substrates, one of the proposed solutions is to combine the sophisticated microelectronics with optoelectronics technologies to achieve Si-based single-chip photoelectric integration circuits with photons as information carriers. Many other processes in Si-based monolithic optoelectronic integration are more mature [1,2,3,4,5], and the greatest limitation is the lack of suitable light sources.

However, Si itself is an indirect band gap semiconductor that cannot be used as an efficient luminescent material directly. In 2000, Pavesi et al. first reported on the nc-Si optical gain and stimulated emission at room temperature, and it is expected to become an efficient luminescent material [6]. Obtaining more suitable Si-based luminescent materials and improving the related fluorescence quantum efficiency has been one of the most difficult tasks in this field for more than two decades [7,8,9,10,11,12,13,14,15,16,17,18,19,20,21,22,23,24,25,26,27,28,29,30,31,32,33,34,35,36,37,38,39,40,41,42].

In recent years, the research on Si-based luminescent materials has been extensive, effectively improving the fluorescence quantum efficiency values. Negro et al. and Atwater et al. obtained up to 7% PL EQE and 59% PL IQE in nc-Si-embedded Si nitride and Si oxide films, respectively [17,18]. In 2014, Zacharias et al. systematically studied the PL properties of nc-Si-embedded SiN_x_O_y_/SiO_2_ multilayer films, and obtained a PL absolute quantum yield (PL QY, or PL EQE) value of up to 19% at room temperature when the SiO_2_ layer was 2 nm thick [26]. However, the current values for the PL QEs of Si-based materials are still low, indicating that they are still far from practical application. Additionally, most of the previous research works focused on Si-based nanostructure materials, while quantum efficiency studies of non-crystalline Si-based compounds are less frequently reported. As a potentially high-efficiency luminescence material in the integration of silicon-based optoelectronics, there has been a great deal of interest in the luminescence properties of a-SiN_x_O_y_ systems and they have been studied in detail [13,14,21,22,23]. However, so far, exciting results from this type of Si-based compound are still lacking.

Based on our previous research [31,32,33,34], in this paper, we systematically demonstrate the high photoluminescence quantum efficiency of a-SiN_x_O_y_ systems in detail. First, we measured the PL absolute quantum yields directly using a calibrated integration sphere at room temperature. Secondly, we used the temperature-dependent PL (TD-PL) spectra of a-SiN_x_O_y_ films for the purpose of estimating the PL IQE values at different temperatures relative to the lowest temperatures. The experimental results showed that the a-SiN_x_O_y_ films had high PL IQE values (~84% at 480 nm emission peak wavelength), compared with those of reported Si-based luminescent thin films. Finally, we also calculated the temperature-dependent PL QYs of a-SiN_x_O_y_ systems, and the related PL mechanisms were discussed.

## 2. Materials and Methods

### 2.1. Material Fabrication

The a-SiN_x_O_y_ films (~300 nm) were deposited on polished and roughened Si or quartz substrates in a plasma-enhanced chemical vapor deposition (PECVD, OXFORD Plasmalab 80Plus, Oxford, UK) system with a silane, ammonia, and nitrogen gas mixture (flow ratio R = NH_3_/SiH_4_) [33]. The fabrication flow ratios and related parameters are listed in Table 1. After fabrication, the samples were subsequently oxidized in situ via oxygen plasma treatment. To obtain high light extraction factors and effectively perform PL EQE measurements, the Si or quartz substrates were roughened using electrostatic sands (particle size range approximately 1–10 μm) before fabrication. To eliminate the light-induced degradation and the PL self-restoring characteristics, post-treatments including improving the density and eliminating weak bonds were carried out on the a-SiN_x_O_y_ films by combining thermal annealing with pulsed laser annealing [34], thus obtaining high performance regarding luminescent stability in a-SiN_x_O_y_ systems.

### 2.2. Characterization of A-SiN_x_O_y_ Thin Films

The films were structurally characterized with a Thermo ESCALAB 250 X-ray photoelectron spectrometer (XPS) with an excitation source of monochromatic Al K-alpha rays (1486.6 eV). The Tauc’s optical gap (E_opt_) under different R conditions was obtained from transmittance measurements at room temperature, which were performed using a Shimadzu UV-3600 spectrophotometer. The steady-state TD-PL properties were measured with a Fluorolo-3 system (Jobin Yvon) and a HP4284 LCR meter in a computer-controlled Delta 9023 oven, using a 75 W Xe lamp (excitation wavelength *λ_exc_* = 250–800 nm) and a He–Cd laser (*λ_exc_* = 325 nm, output power 30 mW) as light sources. As comparative references, controlled a-SiN_x_ films with different flow ratios R were also prepared and the corresponding characteristics were measured under the same conditions. Before we performed all the measurements, we carefully checked and corrected the intensity of the light source, the response of the detector, the influencing factors of sample holders, and the sample’s morphology. All the recorded PL spectra were background-corrected.

### 2.3. PL EQE (PL QY) Measurement Methods

The PL EQE (PL QY) measurements were determined using a calibrated integrating sphere (Jobin Yvon quantum-φ) with a Xe lamp (Xenon short ARC), which was used to excite samples under the excitation wavelength of 375 nm at room temperature. The excited and emission photons were collected by a 1-mm-diameter optical fiber (ocean optics) and detected using a Fluoromax-4 system (Jobin Yvon).

Generally, the PL absolute quantum yield (PL QY) is defined as PL QY =ϕ_emission_/*ψ*_absorption_. Here, *ϕ*_emission_ and *ψ*_absorption_ are the number of photons emitted from the samples and the number of absorbed photons at the excitation wavelength, respectively.

**Figure 1:**
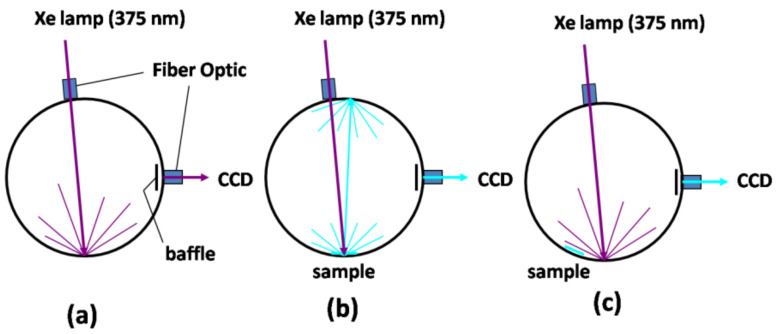
A diagrammatic presentation of the optical paths of the emitted photons from the excitation light source and the a-SiN_x_O_y_ films in the integrating sphere during PL QY measurement processes. (**a**) No a-SiN_x_O_y_ samples; (**b**) the excitation light shines directly onto a-SiN_x_O_y_ samples; (**c**) the excitation light shines onto a-SiN_x_O_y_ samples indirectly.

In this work, we directly measured the PL QY values of a-SiN_x_O_y_ thin films for different R in the calibration integrating sphere. The optical paths of emitted photons from the excitation light source and the a-SiN_x_O_y_ films in the integrating sphere are schematically shown in Figure 1.

The detailed PL QY measurement processes are described as follows:

(a) We directly measured the total incident excitation photons *ψ*_0_ in the integration sphere (schematic diagram in Figure 1a);
(b) When the excitation source was shone directly onto samples, we directly measured the unabsorbed excitation photons *ψ*_1_ and corresponding emitted photons *ϕ*_1_ (schematic diagram in Figure 1b);
(c) When the excitation source was shone indirectly onto samples, we directly measured the unabsorbed excitation photons *ψ*_2_ and corresponding emitted photons *ϕ*_2_ (schematic diagram in Figure 1c).

The PL QY can be given by

(1) $$\mathrm{PL}\;\mathrm{QY}=\frac{\phi_{\mathrm{emission}}}{\psi_{\mathrm{absorption}}}=\frac{\phi_{1}-\left[1-A\right]\times \phi_{2}}{\psi_{0}\times A}=\frac{\phi_{1}\psi_{2}-\psi_{1}\phi_{2}}{\psi_{0}\times \left(\psi_{2}-\psi_{1}\right)},\;\mathrm{and}\;A=1-\frac{\psi_{1}}{\psi_{2}}$$

where A is the film absorbance when the excitation light is directly shone onto the sample.

## 3. Results and Discussion

### 3.1. X-Ray Photoelectron Spectrum (XPS)

**Figure 2:**
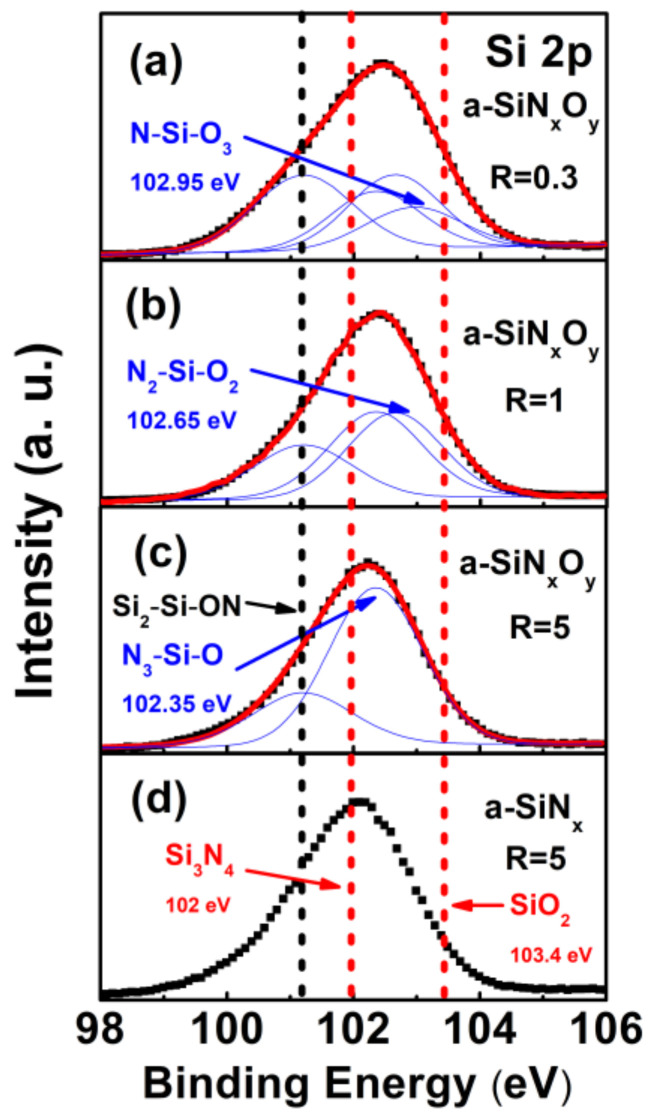
XPS spectra of Si 2p of both a-SiN_x_O_y_ with (**a**) R = 0.3; (**b**) R = 1; (**c**) R = 5, and the controlled a-SiN_x_ films with (**d**) R = 5, which was measured after Ar ion beam sputtering at 90 nm depth. For a-SiN_x_O_y_ films with different R, the Voigt peak fitting results are also shown as a solid line.

We analyzed the chemical composition ratio and bonding configuration of a-SiN_x_O_y_ films under different R by using XPS spectroscopy. Figure 2 represents the XPS spectra of Si 2p of both a-SiN_x_O_y_ under different R and the controlled a-SiN_x_ films, respectively. Before measurement, all sample surfaces were etched for 15 min using an Ar+ beam (Ar+ energy 2000 eV, etch depth ~90 nm). From Figure 2, one can see that the Si 2p peak positions of a-SiN_x_O_y_ films were between the Si 2p peak position of the standard proportion Si_3_N_4_ (102 eV) and SiO_2_ (103.4 eV), which indicated the existence of the N-Si-O bonding configurations in a-SiN_x_O_y_ films [15,16]. We adopted four typical N-Si-O bonding configurations to decompose the XPS spectra of Si 2p, whose binding energies peaked around 101.2 eV (Si–Si_2_ON), 102.35 eV (Si–N_3_O), 102.65 eV (Si–O_2_N_2_), and 102.95 eV (Si–O_3_N) [15,16], and the F.W.H.M. of these sub-peaks were consistent, as shown in Figure 2.

**Figure 3:**
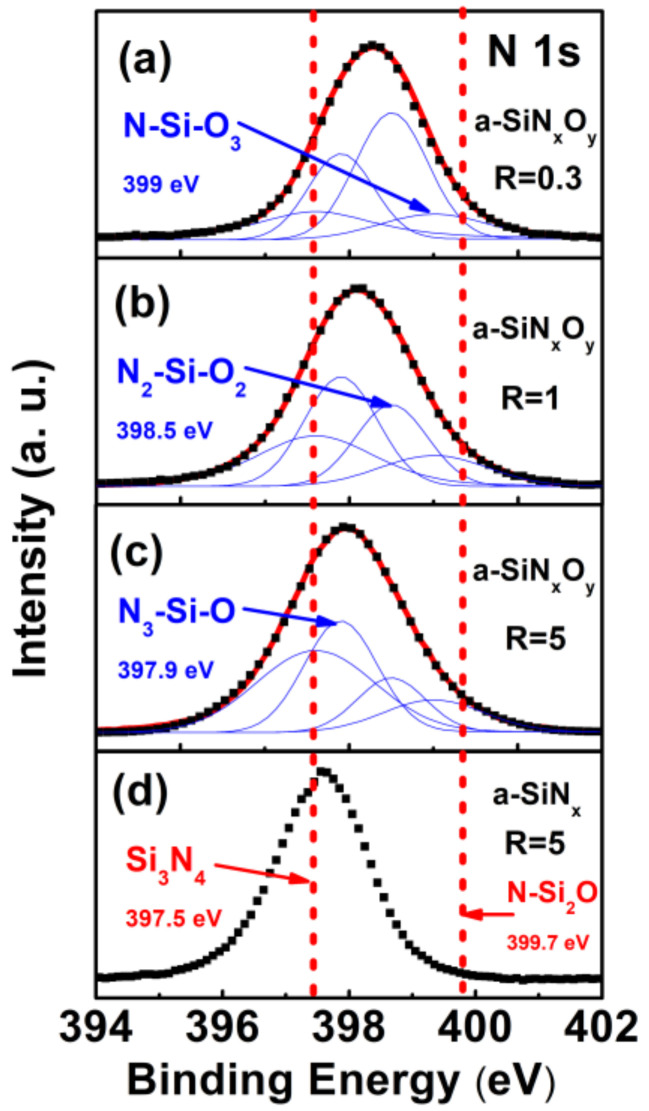
XPS spectra of N 1s of both a-SiN_x_O_y_ with (**a**) R = 0.3; (**b**) R = 1; (**c**) R = 5, and the controlled a-SiN_x_ films with (**d**) R = 5, which was measured after Ar ion beam sputtering at 90 nm depth. For a-SiN_x_O_y_ films with different R, the Voigt peak fitting results are also shown as a solid line.

From the N 1s spectra in Figure 3, we can also observe similar rules. For a-SiN_x_O_y_ films with different R, the binding energies of the N 1s peaks were located between the N 1s peak position of the standard proportion Si_3_N_4_ (397.5 eV) and the N-Si_2_O bonding configuration (399.7 eV), respectively, in correspondence with the binding energies: three types of bonding configuration [15,16], namely N_3_-Si-O (397.9 eV), N_2_-Si-O_2_ (398.5 eV), and N-Si-O_3_ (399 eV).

Therefore, from the variation characteristics of the Si 2p and N 1s binding energy of the XPS spectrum of a-SiN_x_O_y_ films, there was indeed an arbitrary mixed bond configuration of Si, N, and O atoms in the a-SiN_x_O_y_ films: the N-Si-O bonding configuration. We calculated the incorporation concentration of O from the measured Si 2p, N 1s, and O 1s energy peaks, and its value was stable in the range of 2~6% under different R conditions.

### 3.2. PL Emission and Excitation (PLE) Characteristics

**Figure 4:**
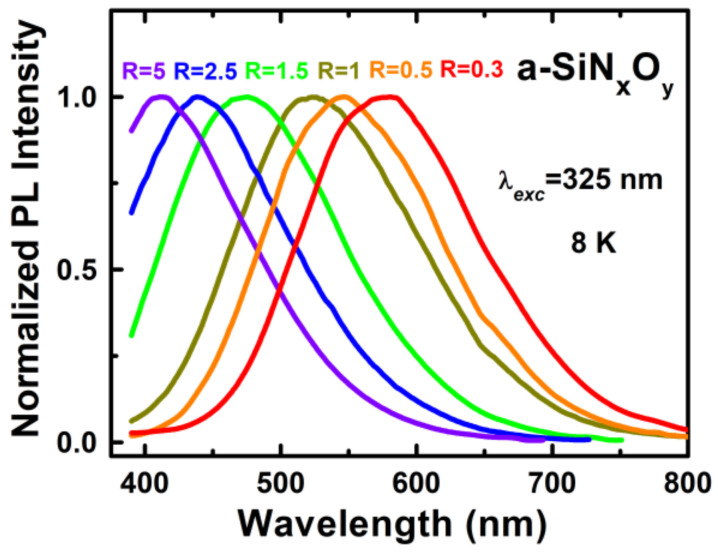
Normalized PL spectra of the a-SiN_x_O_y_ films for various R under the excitation wavelength of 325 nm at 8 K.

To investigate the fluorescence properties of the a-SiN_x_O_y_ films, the PL emission and excitation (PLE) characteristics were measured. Figure 4 shows the normalized PL spectra of the a-SiN_x_O_y_ films for various R under the excitation wavelength of 325 nm at 8 K. It was notable that, by adjusting the NH_3_/SiH_4_ flow ratio, bright tunable light emissions in the visible range from the a-SiN_x_O_y_ samples were achieved and were perceptible by the naked eye. One can see that, with the increasing R values from 0.3 to 5, the PL peaks (E_PL_) showed a blue shift from 582 to 411 nm.

**Figure 5:**
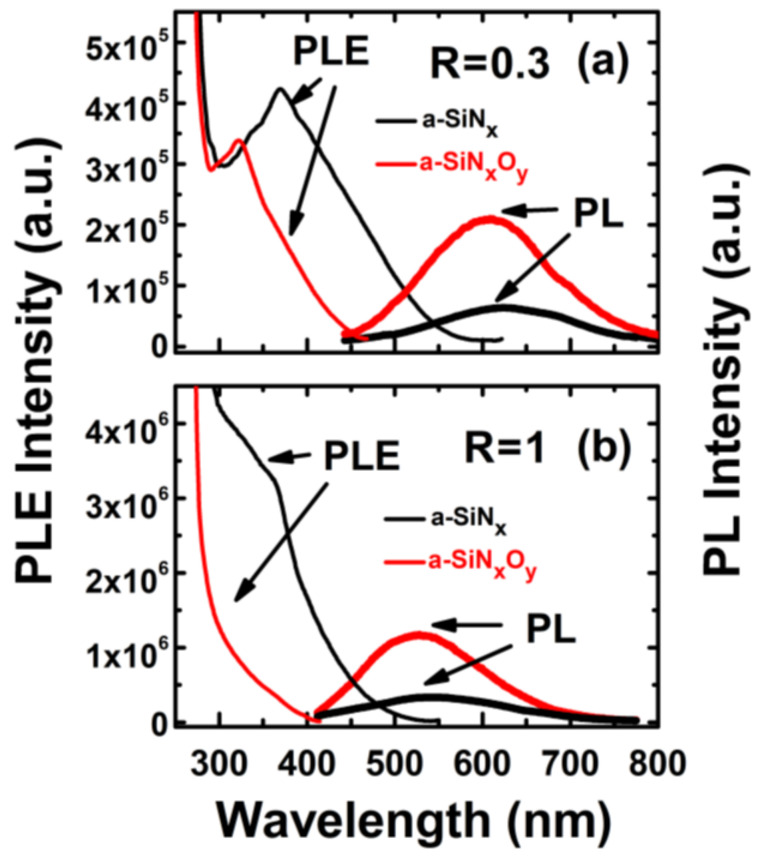
PL and PLE spectra of a-SiN_x_O_y_ and a-SiN_x_ films with (**a**) R = 0.3 and (**b**) R = 1 at room temperature.

Figure 5a,b show the PL and PLE spectra of a-SiN_x_O_y_ films and a-SiN_x_ films at room temperature when R = 0.3 and R = 1, respectively. Under the excitation of a 325 nm He–Cd laser, the PL intensities from a-SiN_x_O_y_ films were significantly enhanced with the incorporation of O, with slightly blue-shifted PL peak positions, compared with those of controlled a-SiN_x_ films for the same R. For a-SiN_x_O_y_ and a-SiN_x_ films, the E_opt_ as well as E_PL_ of the a-SiN_x_O_y_ films could be controlled by the reaction gas with different R. The detailed E_opt_ and E_PL_ values for various R are listed in Table 1. As the flow ratio R increased, we found that both E_PL_ and E_opt_ first monolithically increased (blue-shifted) and then became saturated.

However, from the PL emission spectra, the difference in the structure of the a-SiN_x_ thin films before and after the incorporation of O atoms could not be directly or clearly distinguished. Therefore, we went on to study the PLE spectra of both types of samples. As we can see from Figure 5a,b, both types of films had excitation state band tail structures, but the width of the conduction band tail (E_U_) was different when R was the same. Compared with the controlled a-SiN_x_ films, the Urbach tail energy (E_U edge_, or conduction band tail, E_CB tail_) of a-SiN_x_O_y_ films shrank towards the high energy level, and this represented the lessening of E_U_ with the same R. This was mainly because the Si-O bonds formed in the a-SiN_x_O_y_ film could release more internal stress in the film after the incorporation of O atoms [14], resulting in structural disorder in the a-SiN_x_O_y_ films, which was manifested as a decrease in the band tail width E_U_ and formed a luminescent N-Si-O defect center in the excited state [33]. We measured the luminescence characteristics of other assembled samples under different R and obtained similar results.

### 3.3. The Doping Effect of O Atoms on the Luminescence Characteristics

To further understand the luminescence characteristics of the a-SiN_x_O_y_ films before and after the incorporation of O atoms, we extensively investigated the varied relationships of E_PL_ with the Urbach tail edge (E_CB tail_) and the PL Full Width Half Maximum (F.W.H.M.) with the Urbach tail width (E_U_), respectively.

The typical PLE spectra exhibited the density distribution of luminescent excited states. For the amorphous semiconductor materials, it included extended and localized band tail states, as shown in Figure 5. According to the definition, the Urbach energy edge (Urbach tail edge or conductive band tail edge, E_U edge_ or E_CB tail_) is approximately equal to the PLE threshold energy (the onset portion of the PLE spectrum). The Urbach tail width (E_U_) can be calculated from the equation of demarcation energy (E_C_ or E_opt_) minus E_CB tail_ (E_U_ = E_opt_ − E_CB tail_). The detailed optical parameters of the E_CB tail_ and E_U_ of the a-SiN_x_O_y_ films for various R are listed in Table 1. For comparison, the optical parameters of three controlled a-SiN_x_ samples were also summarized and calculated.

**Figure 6:**
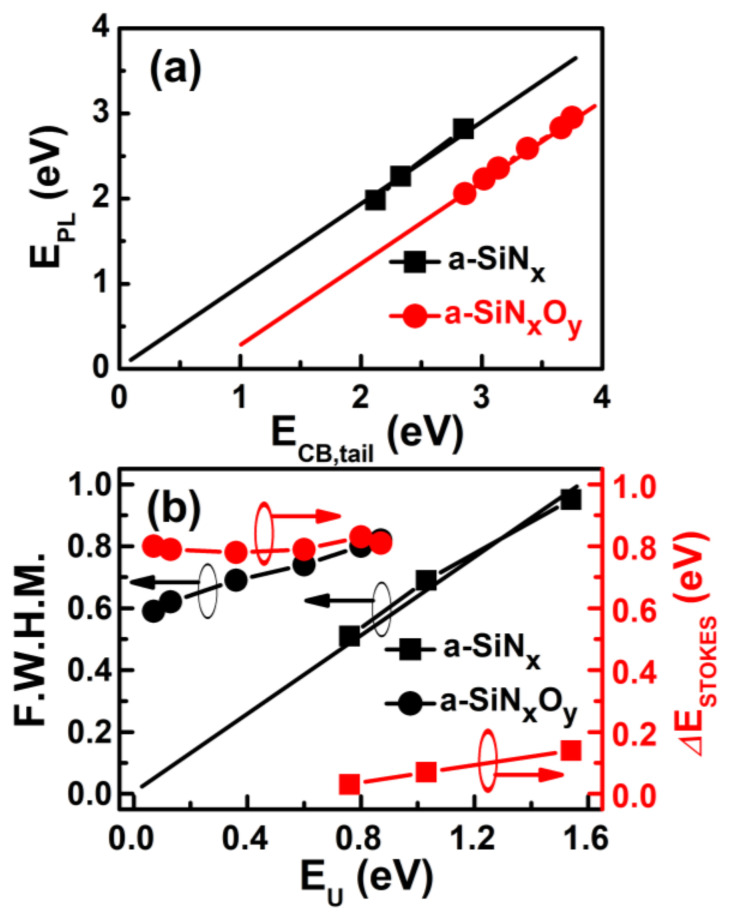
The measured (**a**) E_PL_ vs. E_CB tail_; (**b**) PL F.W.H.M. and ΔE_stokes_ vs. E_U_ of a-SiN_x_O_y_ and a-SiN_x_ films, respectively.

From Figure 6a, for both types of films, E_PL_ was proportional to E_CB tail_, whose level direction shrank when the N content increased. For a-SiN_x_ films, E_PL_ was relatively close to E_CB tail_ (the slope was close to 1), and the straight line of the function between the two nearly passed the origin of the coordinate axes, indicating a typical band-tail-related PL mechanism, whose variation tendencies were similar to previously reported results [8]. However, for a-SiN_x_O_y_ films, we noticed that there was a Stokes shift between E_PL_ and E_CB tail_ (ΔE_stokes_ defined as ΔE_stokes_ = E_U edge_ − E_PL_). As shown in Figure 6b, the E_PL_ positions of a-SiN_x_O_y_ films were fixed at a nearly constant value of approximately 0.8 eV below the conduction band tail level (from 0.75 eV when R = 0.3 to 0.84 eV when R = 5), indicating the PL generated from oxygen-induced localized luminescent defect states, which was pinned under E_U edge_ for various E_opt_. This PL phenomenon is completely different from the previously reported luminescence mechanism of a-SiN_x_O_y_ with oxygen doping greater than 10% [14,21]. For comparison, the a-SiN_x_ films had a ΔE_stokes_ value from 0.03 eV to 0.14 eV for various R, which is also related to the typical band tail PL in a-SiN_x_ systems.

From Figure 6b, we also can see that the relationship between the PL F.W.H.M. and E_U_ is different for both types of materials. The PL F.W.H.M. of a-SiN_x_ thin films is proportional to E_U_, and the function line moves nearly straight past the origin of the coordinate axes. However, for a-SiN_x_O_y_ films, the PL F.W.H.M. has an average width of 0.7 ± 0.1 eV. We further measured the PL behavior of a-SiN_x_O_y_ films at different excited photon energies (E_exc_), and we found that the spectral profile and luminescence peak of the PL spectrum did not change with the change in E_exc_. These are typical defect-state luminescence characteristics. Therefore, we believe that O atoms are incorporated to form defect states in the films, and the PL of a-SiN_x_O_y_ films arises from the luminescent N-Si-O defect centers.

### 3.4. The Absolute PL Quantum Yield Measurements

We directly measured the PL QY values of a-SiN_x_O_y_ samples by using a calibrated integrating sphere under the excitation wavelength of 375 nm at room temperature. It is worth mentioning that when the excitation light does not shine directly on the sample, but on the integrating sphere wall, the sample also absorbs some of the excitation photons diffusely reflected by the integrating sphere wall, as shown in Figure 1c. Thus, the measured total number of excitation photons absorbed by the sample becomes larger, and we should consider the effect of scattered light on the sample and correct the resulting PL QY values directly obtained from the integrating sphere.

**Figure 7:**
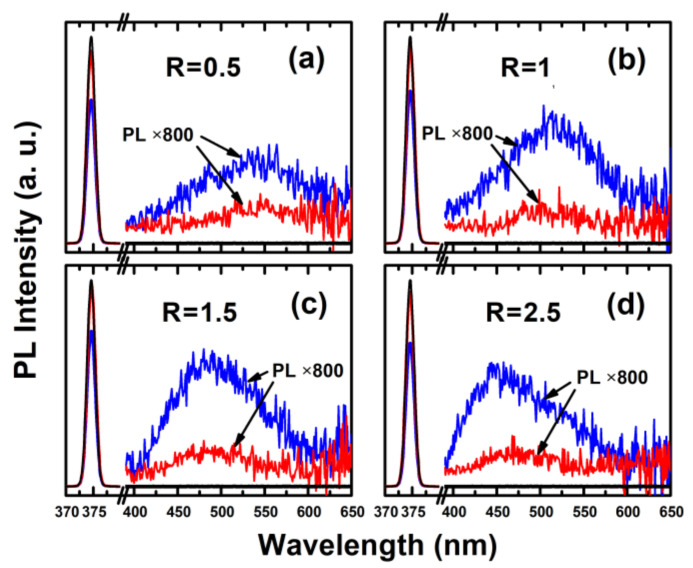
The PL QY measurement processes of the a-SiN_x_O_y_ films with (**a**) R = 0.5; (**b**) R = 1; (**c**) R = 1.5 (Reprinted from ref. [33]); (**d**) R = 2.5. Black line: the total incident excitation photons (**left**). Red line: the unabsorbed excitation photons (**left**) and corresponding emitted photons (**right**) when the excitation source is directly shone onto samples. Blue line: the unabsorbed excitation photons (**left**) and corresponding emitted photons (**right**) when the excitation source is indirectly shone onto samples.

Figure 7 shows the light absorption spectra of the excitation light source and the PL emission spectra of the a-SiN_x_O_y_ films with R = 0.5, 1, 1.5 [33], and 2.5, respectively, both of which are parts of the PL QY measurement processes. By using Equation (1), for the PL peak range of 2.06–2.95 eV, the a-SiN_x_O_y_ films were found to have PL QY values ranging from approximately 2% to 10% in the visible range at room temperature, as listed in Table 2. The PL QY of 10.08% ± 0.95 has been achieved at *E*_PL_ = 2.6 eV, which is higher than those of reported nc-Si-embedded Si-based thin films [17], a-SiN_x_O_y_ films [32], and a-SiC_x_O_y_ films [36].

### 3.5. The Temperature-Dependent PL Internal Quantum Efficiencies

**Figure 8:**
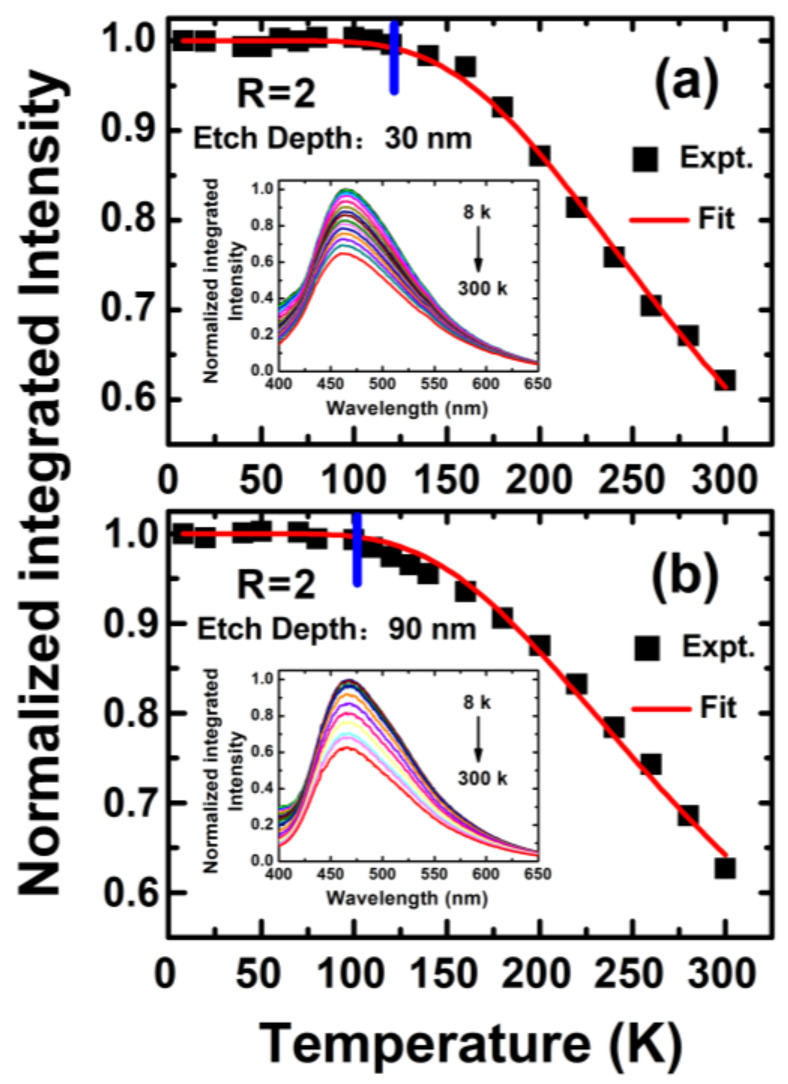
Normalized integrated TD-SSPL intensities of a-SiN_x_O_y_ thin films (R = 2) with surface etch depths (**a**) 30 nm and (**b**) 90 nm, respectively. Red line: Theoretical fitting results of the TD-SSPL intensities of a-SiN_x_O_y_ thin films; Blue line: The temperature inflection points of luminescence thermal quenching. The inset shows the related measured TD-SSPL spectra.

Temperature-dependent steady-state photoluminescence spectroscopy (TD-SSPL) is usually used to determine and verify the photoluminescence radiation recombination mechanism, and the PL internal quantum efficiencies of the samples can also be studied and estimated from the TD-SSPL measurements. In general, radiation recombination at low temperatures dominates the recombination processes, which means that the PL internal quantum efficiency is close to 100% [8,17,20]. The samples were placed in a Dewar apparatus and heated by precisely controlling the compressed air cooling and heating power supply to achieve different test temperatures. Figure 8 shows the TD-SSPL spectra of a-SiN_x_O_y_ thin films with R = 2 under variable temperatures (temperature range from 8 K to 300 K). Before measurement, the sample surfaces were etched in situ via Ar+ sputtering with different etch times, as shown in Figure 8a,b (etch depths are 30 nm and 90 nm, respectively). One can see that, at different temperatures, the PL curve shapes and the PL peak positions do not change significantly, indicating that the PL arises from the luminescent defect centers, and the radiation recombination processes are different from the recombination mechanism through the band-tailed states.

In this work, we used the thermal ionization models [17] to simulate and estimate the PL internal quantum efficiency under different temperatures $I_{PL}\left(T\right)=\frac{I_{PL}\left(T_{0}\right)}{1+\tau exp{\left(-E_{A}/kT\right)}^{}}$. Here, *I_PL_*(*T*_0_) and *I_PL_*(*T*) are the integrated PL intensities of the samples at low temperature range *T*_0_ and at a specific test temperature *T*; τ is inversely proportional to the radiation recombination rate, and E_A_ is the activation barrier energy. Since electron–hole pairs dominate in radiative recombination at low temperatures, after normalizing the TD-SSPL spectra, the *I_PL_*(*T*) curves and the PL IQE at any given temperature can be well fitted according to the thermal ionization model, as shown in Figure 8. From the fitted *I_PL_*(*T*), τ ≈ 10 and E_A_ ≈ 62 meV are obtained, which are similar to previously reported results [17]. Notably, we found that the PL intensities of all samples did not change before 80 K. After 120 K, there was significant thermal quenching, indicating an increase in the non-radiative compounding factors.

At the same time, we found an interesting phenomenon wherein the *I_PL_*(*T*) of the sample after in situ etching at a 30 nm depth had significant thermal quenching after 120 K, while, when etching at a 90 nm depth, the inflection point of the *I_PL_*(*T*) was advanced, and the *I_PL_*(*T*) began to decrease significantly at 90 K. The reason is that oxygen is mainly doped and distributed on the surfaces of a-SiN_x_O_y_ samples as an impurity [34]. On one hand, the N-Si-O bonding phase is more stable than the N-Si bonding; the PL attenuation phenomenon of the sample after in situ etching at a 30 nm depth is improved compared to that of 90 nm samples. On the other hand, due to the luminescence of a-SiN_x_O_y_ film samples, mainly derived from the luminescent defect center related to N-Si-O, with the increase in the etching time, the concentration of N-Si-O defect states decreases and the PL light efficiencies decrease slightly. Therefore, this phenomenon further confirms, from another perspective, that the formation of the N-Si-O bonding configuration can not only improve the luminescent stability, but also improve the luminescent efficiency.

We also measured the TD-SSPL properties of a-SiN_x_O_y_ films with different R ratios (R = 0.3, 1, 1.5, 5); the related PL IQE values are listed in Table 2. For a-SiN_x_O_y_ with R = 1.5, the PL IQE values at room temperature fitted from TD-SSPL measurements are approximately 84.1%, which are consistent with the calculated PL IQE (ε ~ 83.6%) obtained from the direct measurement of PL QYs, and much higher than those of reported nc-Si-embedded Si-based thin films [17,18], a-SiN_x_O_y_ films. [20,21,22,23,32], and a-SiC_x_O_y_ films [36].

### 3.6. The Temperature-Dependent PL External Quantum Efficiencies

Finally, we studied the temperature-dependent PL external quantum efficiencies (PL QY) of a-SiN_x_O_y_ samples with various R. Combined with the PL QY values directly measured from the integrating sphere at room temperature (for each PL QY with various R measured at RT, we should take the average value before calculation), and the PL integrated intensities under different measurement temperatures obtained from TD-SSPL measurements, we could directly calculate the PLQY values of a-SiN_x_O_y_ samples with various R under different temperatures by using the formula $\eta \left(T\right)=\frac{I_{PL}\left(T\right)}{I_{PL}\left(300\;K\right)}\;\eta \left(300\;K\right)$. Here, *I_PL_*(*T*) and *I_PL_*(300 K) represent the PL-integrated intensities at a specific temperature T and room temperature, respectively. *η*(*T*) and *η*(300 K) represent the PL QY values of a-SiN_x_O_y_ samples at the specific temperature T and room temperature, respectively.

**Figure 9:**
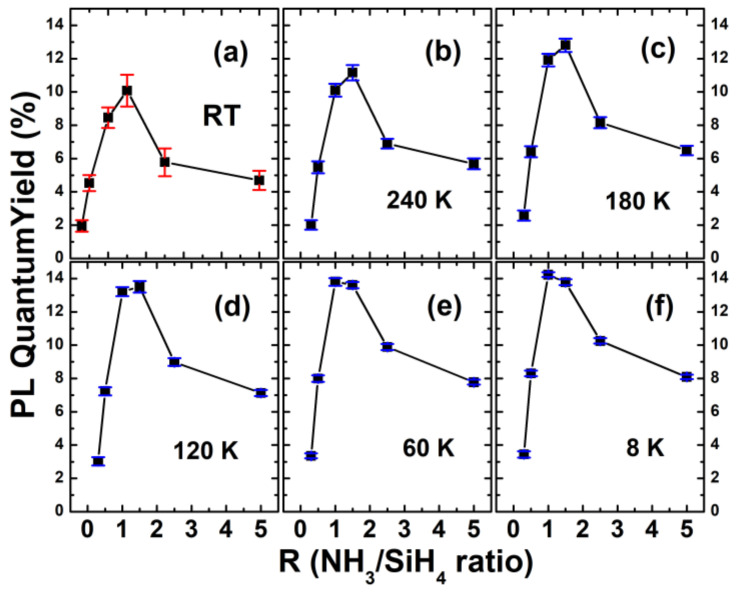
The PL QY of a-SiN_x_O_y_ samples with various R under measurement temperatures of (**a**) RT; (**b**) 240 K; (**c**) 180 K; (**d**) 120 K; (**e**) 60 K; (**f**) 8 K. Red and blue lines show the error bars of the temperature-dependent PL QY values.

Figure 9 shows the PL QY values of a-SiN_x_O_y_ samples with various R under different measurement temperatures (8 K, 60 K, 120 K, 180 K, 240 K, and RT, respectively). The detailed temperature-dependent PL QY values for various R are listed in Table 2. One can see that, for various R, the variation tendency of the PL QY values was consistent with the variation tendency of N_x_ defect densities [32], indicating that luminescent N_x_ defects were responsible for the high PL internal quantum efficiencies in our a-SiN_x_O_y_ systems. The a-SiN_x_O_y_ samples with R = 1 had a PL QY value of approximately 14.23% ± 0.15% at the lowest temperature range (~8 K), as shown in Figure 9f.

## 4. Conclusions

In this study, we systematically researched the PL quantum efficiencies of luminescent a-SiN_x_O_y_ systems at different temperatures in the visible range. From a calibrated integration sphere, we directly measured the PL QYs of a-SiN_x_O_y_ and they ranged from approximately 2% to 10% at room temperature. Then, we calculated the PL IQE values by using temperature-dependent PL spectra, and obtained that the a-SiN_x_O_y_ films had high PL IQE values (~84% at 480 nm emission peak wavelength) compared to those of reported Si-based luminescent thin films. We found that the formation of the N-Si-O bonding configuration can not only improve the luminescent stability, but also improve the luminescent efficiency. Lastly, we also calculated the temperature-dependent PL QYs of the a-SiN_x_O_y_ systems.

## Figures and Tables

**Table 1:** Summary of the fabrication parameters, E_opt_, E_PL_, E_U_, E_CB tail_, F.W.H.M., and ΔE_stokes_ of a-SiN_x_O_y_ systems at room temperature.

R	SiH_4_:N_2_ (5%:95%) (SCCM)	NH_3_ (SCCM)	E_opt_ (eV)	E_PL_ (eV)	E_U_ (eV)	E_CB tail_ (eV)	F.W.H.M. (eV)	ΔE_stokes_ (eV)
0.3	80	1	2.93	2.06	0.07	2.86	0.59	0.8
0.5	80	2	3.15	2.23	0.13	3.02	0.62	0.79
1	80	4	3.5	2.36	0.36	3.14	0.69	0.78
1.5	80	6	3.98	2.59	0.6	3.38	0.74	0.79
2.5	80	10	4.46	2.83	0.8	3.66	0.8	0.83
5	80	20	4.62	2.95	0.87	3.75	0.82	0.81

**Table 2:** Summary of the PL IQE values (average) at room temperature and the temperature-dependent PL QY values (average) of a-SiN_x_O_y_ systems.

R	PL IQE (%) (RT)	PL QY (%)					
		RT (Measured)	240 K	180 K	120 K	60 K	8 K
0.3	25.5	1.95	2.02	2.57	3.03	3.35	3.44
0.5	42.1	4.53	5.47	6.41	7.23	7.99	8.31
1	72.7	8.46	10.10	11.92	13.21	13.81	14.23
1.5	84.1	10.08	11.16	12.81	13.51	13.61	13.79
2.5	45.6	5.78	6.90	8.16	8.98	9.89	10.25
5	35.4	4.69	5.69	6.48	7.13	7.77	8.09

## Data Availability

The data presented in this study are available on request from the corresponding author.
